# Influenza vaccination among children with idiopathic nephrotic syndrome: an investigation of practices

**DOI:** 10.1186/s12882-019-1240-2

**Published:** 2019-02-25

**Authors:** Roman Klifa, Julie Toubiana, Alizée Michel, Nathalie Biebuyck, Marina Charbit, Laurence Heidet, Saoussen Krid, Pauline Krug, Rémi Salomon, Olivia Boyer

**Affiliations:** 1 Néphrologie Pédiatrique, Centre de Référence du Syndrome Néphrotique Idiopathique de l’enfant et l’adulte, Assistance Publique-Hôpitaux de Paris, Hôpital Necker-Enfants Malades, Université Paris Descartes, Sorbonne paris Cité, 149 rue de Sèvres, 75015 Paris, France; 2 Pédiatrie Générale et Infectieuse, Assistance Publique-Hôpitaux de Paris, Hôpital Necker-Enfants Malades, Université Paris Descartes, Sorbonne paris Cité, Paris, France

**Keywords:** Influenza, Vaccination, Idiopathic nephrotic syndrome, Practices, Efficacy, Safety

## Abstract

**Background:**

Annual influenza vaccination is recommended for all children with idiopathic nephrotic syndrome (INS) in France. Consequently, the Social Security automatically sends prescriptions to all patients suffering from a chronic disease. The aim of this study was to evaluate the follow-up to these recommendations.

**Methods:**

We conducted a monocentric retrospective investigation of practices. We included all children with steroid-sensitive INS in remission who attended our clinics from January 1st 2015 to January 1st 2017, resided in France and had a valid phone number. Data were collected from May 2017 to June 2017 through a phone interview and review of clinical charts.

**Results:**

75 patients met the inclusion criteria. The parents of 57 children could be reached by phone and agreed to participate to the survey. 35/57 (61.4%) declared having received a prescription during the 2016–2017 campaign. Only 14 children (24.6%) were vaccinated. 17/43 (39.5%) parents of unvaccinated children had concerns about the safety of the vaccine, 16/43 (37.2%) were not aware of the recommendations, 5/43 (11.6%) had been recommended by their physician not to vaccinate their child, 3/43 (7%) forgot to have them vaccinated and 2/43 (4.6%) reported no reason. 13/43 (30%) unvaccinated children presented a relapse during the flu season - 2/13 during an influenza-like illness - whereas 1/14 (7%) immunized children presented a relapse during the six months of post-vaccination follow-up. Relapse rates were not increased in vaccinated children compared to unvaccinated children (*p* = 0.15), nor in the 6 months following vaccination compared to the 6 months prior (1/14 vs 5/14, *p* = 0.20).

**Conclusions:**

1) < 2/3 patients were properly prescribed the recommended yearly influenza vaccination at our center 2) only 1/4 were vaccinated and most of their parents were misinformed. Physicians must be aware of this and should make every effort to better inform their patients on the risks of flu illness and the benefits and safety of the vaccination.

**Electronic supplementary material:**

The online version of this article (10.1186/s12882-019-1240-2) contains supplementary material, which is available to authorized users.

## Background

Idiopathic nephrotic syndrome (INS) is the most prevalent glomerular disease in children with an incidence ranging from 1 to 4/100.000 children/year [1]. It is characterized by massive proteinuria and hypoalbuminemia. An immunological origin has long been postulated, although the precise mechanisms underlying the disease remain under debate. Many of these children subsequently relapse and require long-term steroid therapy and/or steroid-sparing agents. Immunogenic stimuli such as viral infections or vaccine shots have been incriminated for triggering off relapses [2, 3]. On the contrary some viral infections like measles may induce long-lasting remissions [4].

The flu is a common contagious disease caused by primary influenza A or B infection. Worldwide, annual epidemics result in approximately 1 billion cases of influenza, in about three to 5 million cases of severe illness, and in about 250,000 to 500,000 deaths [5]. In 2016–2017’s winter the number of consultations for influenza-like illness during the epidemic was estimated to 2.3 millions in France in which the proportion of patients under 15 years of age was 42%, the highest rate since 2011 [6].

In healthy children, the systemic symptoms are usually moderate and serious complications are unusual. Symptoms typically include the sudden onset of lasting high fever, cough and muscle aches. Other common symptoms include headache, chills, loss of appetite, fatigue and sore throat. Nausea, vomiting and diarrhea may also occur, especially in children. Most people will recover within a week or 10 days, but some are at greater risk of more severe complications, including pneumonia, encephalitis, myocarditis, and death [7]. Indeed, the highest risk of complications occurs among pregnant women, children aged from 6 to 59 months, people above 65 years, patients with chronic disease (including kidney diseases) or receiving immunosuppressive treatment, and obese subjects [5].

To prevent flu disease and its complications, the World Health Organisation recommends vaccination for at-risk groups such as children from 6 months to 5 years or individuals with chronic medical conditions [5]. In France, the High Council for Public Health (HCSP) recommends annual influenza vaccination in all children with INS. To this end, the French Health insurance sends yearly, as soon as the vaccine has been released, a flu-vaccine prescription to the patients’ homes. The aim of this study was to evaluate the follow-up to these recommendations.

## Methods

We conducted a monocentric retrospective investigation of practices on children followed in the pediatric nephrology unit at Necker Children’s Hospital, Paris, France. We included all consecutive children (1–18 years) with steroid-sensitive INS in remission for at least 3 months, who attended our pediatric nephrology clinics for the past 2 years, who resided in France, and had a valid phone number. Steroid-sensitive INS was defined by achievement of complete remission (negative proteinuria on 2 consecutive days) within 1 month of daily prednisone therapy at 60 mg/m^2^ ± 3 methylprednisolone pulses [8]. Data were collected from May 2017 to June 2017 for the 2016–2017’s flu season through a phone interview of the patient’s parents/legal guardians (Additional file 1) and data were gathered from patients’ chart. A single operator who did not know the patients and their families collected the data. Patients who could not be reached by phone or did not agree to participate were excluded from the study. In this retrospective survey, the flu was defined only by the symptoms of influenza-like illness (ILI) (i.e., temperature ≥ 38 °C for several days, chills, muscle aches and either cough or sore throat). The relapse was defined by (i) a proteinuria ≥3+, for three consecutive days and (ii) the need for intensified steroid therapy. Transient proteinuria was not considered as a relapse.

During the 2016–2017’s flu season, the available vaccine in France was an intramuscular inactivated trivalent vaccine with 2 viral strains of influenza type A (H1N1 and H3N2) and 1 viral strain of influenza type B. For the pediatric population, the vaccination strategy depends on the age. Between six to 35-month-old children: 2 half dose (0.25 ml) of vaccine are given for primary immunization and only 1 half dose for booster. Between three to eight-year-old children: 2 doses (0.5 ml) within a month are administered for primary immunization and 1 dose for booster. Above 9-year-old children only one dose is given. Each dose was given by intramuscular injection. Each season the vaccination campaign begins in October and ends in January.

The study was approved by the local institutional review board, declared to the Information Technology and Liberty Commission (CNIL # 2105091 v0) and conducted in accordance with the declaration of Helsinski.

Analyses were performed using Prism software (GraphPad Software). Results are expressed as mean ± SD or median (range). Continuous data were tested for normal distribution using Kolmogorov-Smirnov test. Normally distributed data were analysed with the Student t test. Non-parametric data were analysed with the Mann-Whitney U test. Frequencies and contingency tables were analysed using the Fisher’s exact two-tailed test. Statistical significance was set at *p* < 0.05.

## Results

**Fig. 1:**
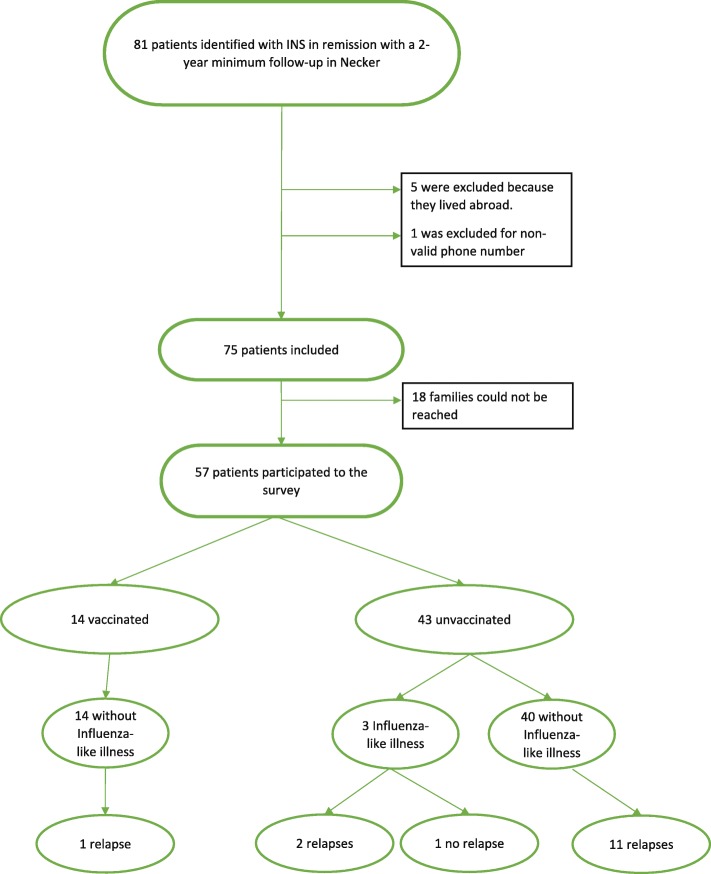
Flow chart

Eighty-one patients followed for INS in the past 2 years were identified. Seventy-five met the inclusion criteria. Fifty-seven parents/legal guardians could be reached by phone; all of them agreed to participate and were included in the study (Fig. 1). The characteristics of the patients are reported in Table 1. The median age at inclusion was 12.8 years and the sex ratio (M/F) was 3:1.

**Table 1:** Patients’ clinical characteristics

Patients’ characteristics	Total *N* = 57	Vaccinated *N* = 14	Nonvaccinated *N* = 43	*P* value
Age (years)				
Median [range]	12.8 [5-18]	13.3 [6-19]	12.4 [6-18]	0.46
Gender				
Boys (%)	39 (70)	11 (78)	28 (65)	0.51
Girls (%)	18 (30)	3 (22)	15 (35)	
Age at the time of diagnosis (years)				
Median [range]	3.3 [1-7]	3 [1-6]	3.4 [1-7]	0.60
Duration of illness (years)				
Median [range]	9.05 [3-16]	9.78 [3-16]	8.72 [3-16]	0.12
Current immunosuppressive treatment	42 (74)	11 (78)	31 (72)	0.73
Prednisone	9	1	8	0.43
Prednisone + tacrolimus	1	0	1	0.22
Prednisone + mycophenolate	15	6	9	1
Prednisone + ciclosporine	3	1	2	1
Prednisone + levamisole	2	0	2	1
Prednisone + tacrolimus + mycophenolate	5	1	4	1
Rituximab	1	1	0	0.25
Mycophenolate	6	1	5	1
No Treatment	15 (26)	3 (22)	12 (28)	0.89
Relapses				
During the six months prior to vaccination and/or flu season	18 (31)	5 (36)	13 (30)	0.74
During the six months following vaccination and/or during flu season	15 (26)	1 (7)	13 (30)	0.31

Twenty-seven families (47.4%) were not aware of the recommendations before this survey. Eighteen children (31.5%) had been vaccinated annually against the flu in the years preceding the study.

Thirty-five parents (61.4%) declared having received a prescription from the French Social Security or from their physician during the 2016–2017 flu season. Fourteen patients in total (24.6%) and 37% of those who had received the vaccine prescription were vaccinated during the campaign, all with an inactivated vaccine (intramuscular injection).

The clinical characteristics did not statistically differ among children who did or did not receive the vaccine (Table 1).

Among the 43 parents of unvaccinated children, 17 (39.5%) had concerns about the vaccine’s safety, 16 (37.2%) were not aware of the recommendations, 5 (11.6%) had been told by their physician (general practitioner, pediatrician or nephrologist) not to immunize their child, 3 (7%) had forgotten to have their child vaccinated before the end of the flu season, 2 (4.6%) reported no reason for non-vaccination.

Among the 43 unvaccinated patients, three (7%) presented an influenza-like illness (Fig. 1). Two of them experienced INS relapse a few days after the beginning of the symptoms. In addition, 11/43 patients presented a relapse during the following 6-month flu season period. Thus, in total, 13/43 (25.5%) unvaccinated patients presented a relapse during the flu season period. Relapse rates were not significantly different between patients without influenza-like illness (*n* = 13/54) and with influenza-like illness (*n* = 2/3, *p* = 0.16).

Conversely, none of the 14 immunized children presented any influenza-like illness and only one (7%) relapsed 15 days following vaccination. Another child presented transient proteinuria 5 months following vaccination. The relapse rate did not significantly differ between vaccinated and unvaccinated children (*p* = 0.15). Moreover, the relapse rate of vaccinated children did not differ between the 6 months preceding and the 6 months following vaccination (5/14 vs 1/14, *p* = 0.20). The same result was observed in the unvaccinated group during the corresponding 6-month period (13/43 vs 13/43, *p* = 1).

At the end of the phone questionnaire, the parents were informed of the recommendations and 31 (54%) parents declared considering having their child vaccinated against the flu in the following year in any case, 13 (23%) others stated they would have their child vaccinated as long as their general practitioner/pediatrician would recommend it as well. The last 13 parents (23%) stated that they would still refuse to vaccinate their children for the next seasons.

## Discussion

This study shows that only a quarter of patients followed for INS at our institution were vaccinated against the flu during the 2016–2017 season, whereas at least 77% of the families would consider having their child vaccinated if their physicians recommended it for the next flu seasons. Among the parents of unvaccinated children, 39% had concerns about the safety of the vaccine, some of whom because of their physicians’ recommendations, but most of them because of a lack of information.

However, these results must be treated carefully. Indeed, the retrospective nature of this study may have biased the data. In this retrospective survey, the diagnosis of influenza-like illness was based on signs and symptoms, as recommended for outpatients. Therefore, some of these influenza-like illnesses may be caused by another virus. The Center for Disease Control (CDC) reported only 54.6% PCR positivity among US patients diagnosed with influenza-like illnesses during the 2017–2018’s winter [9].

In the present study, 2 out of the 3 INS patients with an influenza-like illness relapsed a few days later. In children with INS, viral infections may trigger off a relapse or may be severe with potential pneumococcal infection [10]. Therefore, influenza vaccination has been recommended for children with INS by the French High Council of Public Health since 2000. Larger indications exist around the world: in the United Kingdom, a yearly influenza vaccination is recommended for all children between 2 and 9 years of age [11]. In the USA, the CDC recommends yearly influenza vaccination for every person aged 6 months or more [12]. For the 2016–2017’s season the CDC has recommended to use only the inactivated vaccine, the only one available in France this season, due to the lack of efficiency of the live attenuated one [13]. In Canada, influenza vaccination is recommended for all individuals aged 6 months and older [14].

A decline in vaccination trust has impaired vaccination programs this last decade. Such decline is illustrated by the so-called revival of antivaccination movements [15–17]. The high rate of flu vaccine mistrust observed in our study is in line with the general vaccine mistrust in France, a country with historical difficulties regarding mistrusts towards vaccination [18, 19]. We showed that, despite the recommendations, 39.5% of the parents of non-vaccinated children did not trust the safety of the vaccine and that only one fourth of the patients of the present study were vaccinated against the flu. For the 2016–2017 season, the vaccination rate of the population targeted by the recommendations was 46% which is 1.84 times higher than the vaccinal coverage of our INS patients [20].

As mentioned above, the main reason for non-vaccination in the present study was vaccine mistrust, followed by a lack of information and, in some cases, a recommendation from their physician (general practitioner, pediatrician or nephrologist). This is consistent with the literature, in which the main reason for this opposition is also vaccine mistrust. There are also heterogeneous vaccination practices among physicians. Indeed Pulcini et al. showed that the personality of the general practitioner may be decisive on vaccination behaviour [21]. Some physicians recommended not to immunize some of the patients. We did not know if this was because of an acute event such as an infection at the time of appointment or if it was for fear of relapses. Nevertheless, it remains crucial to insist on the importance of influenza vaccination among our patients.

The effectiveness of influenza vaccination can vary considerably from year to year due to the viral strains, or the immunization programs [22–25]. For the 2016–2017’s season, according to the CDC’s data, the overall vaccine effectiveness was 42%. More precisely the effectiveness was 61% among the patients between 6 months to 8 years of age, and 35% between 9 to 17 years of age [26]. Recently Flannery et al. published the first study using laboratory-confirmed outcomes to investigate influenza vaccine effectiveness [27]. They showed an estimated influenza vaccine effectiveness of 65% (95% CI, 54 to 74%) against laboratory-confirmed influenza-associated deaths among children. This effectiveness was lower in children with underlying medical conditions 51% (95% CI, 31 to 67%), but protection remained significant.

Data on influenza vaccine efficacy in INS are scarce. In their study in 1979, Sheth et al. showed for the first time that INS patients could be effectively immunized against influenza. 83% (25/30) of their patients with renal disease (predominantly INS) had a sufficient antibody titer 1 year after vaccination that was similar to the control group. Poyrazoglu et al. showed an adequate IgG titer 1 and 6 months after influenza A immunization in 19 children with INS [28].

Serological efficacy in INS has also been reported in small series with other vaccines such as VZV-vaccine which induces a lasting antibody response with titers comparable to those of healthy control 2 years later [28, 29]. Ulinski et al. demonstrated a good serological response to the 23-pneumococcal vaccine on high-dose steroids in 43 with INS in remission or in relapse [10] This was subsequently demonstrated with the 13-valent pneumococcal conjugate [30] and is consistent with another series of 29 children with INS who developed adequate antibody titers after booster immunization with 7-valent pneumococcal conjugate vaccine [31]. However, there are no available data on long-term clinical efficacy.

In the present study, patients’ parents/legal guardians reported no flu-like illness in the vaccinated group. However, these results are to be taken with caution since this study was not designed to evaluate the efficacy of influenza vaccination or vaccine-induced disease.

Older publications and a few case reports support the idea that immunizations through vaccination may cause, exacerbate or precipitate relapses in INS. In their study in 1979, Sheth et al. showed that one patient had a minor post-vaccination cold illness and relapsed within days after vaccination [32].

In 2003 *Abeyagunawardena* et al. reported patients with INS among whom 45.7% had relapses “attributable to vaccination” against the Meningococcal C vaccine [33]. This study may be biased due to its retrospective nature. Moreover, it compared the relapse rate in the year preceding vaccination to that of the year following vaccination, although vaccine-induced relapses are expected to occur in the first few weeks following administration. In another small heterogeneous series of 41 children not designed to study the effect of vaccination on NS relapses, Yildiz et al. observed an increased relapse rate from 0.12 ± 0.19 to 0.4 ± 0.12 relapse/month in the month following hepatitis B vaccination (*p* = 0.002) [34]. However, the authors recommend vaccinating children with INS against hepatitis B in endemic regions.

Conversely, all the subsequent publications reported that the vaccination with the VZV, influenza or pneumococcal vaccines seems safe in patients with INS without any significant increase of relapses [29, 31, 35]. For instance, Taylor et al. did not observe any increased risk of relapse of INS after vaccination against Meningococcal C in a cohort of 54 patients [35]. Our study is in accordance with this growing body of evidence regarding the safety of the vaccine (Additional file 2) [28, 29, 31, 35]. Indeed, we did not observe an increased risk of vaccine-induced relapse of INS (1/14 (7%) relapses in the 6 months after vaccination versus 5/14 (36%) in the 6 months before vaccination *p* = 0.20), even though our study was not designed to evaluate the safety of vaccination and the risk of vaccine-induced relapse.

The relapse rate in children who did not present an influenza-like illness seems increased in patients who did not receive the vaccine 11/40 (28%) compared to vaccinated children 1/14 (7%), but the difference is not significant (*p* = 0.15). We do not believe that vaccination has a flu-independent protective effect on the relapse-risk. This trend may due to a selection bias. Moreover, the parental mistrust in vaccination may be associated to a poorer adhesion to other NS treatments in the group of unvaccinated children.

Moreover, there was no significant side effect of the vaccines in the above-mentioned publications. The only concern is with the live vaccines like the VZV or measles for which a steroid therapy less or equal to 2 mg/kg/day is recommended for safety and efficacy [29, 36]. In the present survey, no vaccinated child presented any vaccine-induced influenza-like illness and parents reported no significant side effect.

## Conclusion

This practice survey shows that fewer than 2/3 patients are properly prescribed the recommended yearly influenza vaccination at our center. Only 1/4 of the patients were vaccinated and most of their parents were misinformed. Relapse rates were not increased in vaccinated children compared to unvaccinated children (*p* = 0.15), nor in the 6 months following vaccination compared to the 6 months prior (1/14 vs 5/14, *p* = 0.20).

Pediatric nephrologists and all physicians following INS patients must be aware of this and should make every effort to better inform their patients and parents of the risks of flu illness and of the benefits and safety of the vaccination. To improve practices, we intend, from now on, to send a personal letter of information to each INS patient’s family during the vaccination campaign along with a prescription to reinforce oral recommendations provided during the consultations and Public Health vaccination programs. Besides, recently, 11 vaccines became mandatory in the vaccinal schedule in France. Such a change could probably help to modify French population mistrust of vaccines.

## Additional files

Additional file 1: **Table S1.** Patient questionnaire. (DOCX 70 kb)

Additional file 2: **Table S2.** Review of the literature: (“Nephrotic Syndrome”[Mesh]) AND “Vaccines”[Mesh] AND “Child”[Mesh], limited to 15 years with exclusion of articles written in other languages than English. (DOCX 103 kb)

## Abbreviations

CDC: Center for disease control
HCSP: High Council for Public Health
INS: Idiopathic nephrotic syndrome
