# Supplementary material for: Influenza vaccination among children with idiopathic nephrotic syndrome: an investigation of practices
Source: BMC Nephrol. 2019 Feb 25;20:65. doi: 10.1186/s12882-019-1240-2 (PMC6388483; doi:10.1186/s12882-019-1240-2)
Supplement: Supplementary file 1 — Table S1. Patient questionnaire. (DOCX 70 kb) [file 12882_2019_1240_MOESM1_ESM.docx]

**Supplemental Table 1: Questionnaire**

| Questions | Yes | No | Commentary |
| --- | --- | --- | --- |
| 1. Is your child’s vaccinal calendar in order? |  |  |  |
| 1. Did you receive a prescription for a flu shot from the national Social Security? |  |  |  |
| 1. Do you vaccinate your child every year against the flu? |  |  |  |
| 1. Did your child get a flu shot this year? |  |  |  |
| 1. If No Why? |  |  |  |
| 1. If Yes Why ? |  |  |  |
| 1. Did he/she present a flu-like syndrome this year? If yes did he/she stay at home or was he/she hospitalized? |  |  |  |
| 1. Did he/she had a relapse during the six months following the shot or during the last 6 months? |  |  |  |
| 1. Which treatment was prescribed to your child in the last 6 months? |  |  |  |
| 1. Did you know that the High Council of Public Health recommend yearly influenza vaccination for patients with nephrotic syndrome? |  |  |  |
| 1. Will You have your child get a flu-shot next year? Why ? |  |  |  |
