# Supplementary material for: Influenza vaccination among children with idiopathic nephrotic syndrome: an investigation of practices
Source: BMC Nephrol. 2019 Feb 25;20:65. doi: 10.1186/s12882-019-1240-2 (PMC6388483; doi:10.1186/s12882-019-1240-2)
Supplement: Supplementary file 2 — Table S2. Review of the literature: (“Nephrotic Syndrome”[Mesh]) AND “Vaccines”[Mesh] AND “Child”[Mesh], limited to 15 years with exclusion of articles written in other languages than English. (DOCX 103 kb) [file 12882_2019_1240_MOESM2_ESM.docx]

Table 2: Review of the literature: ("Nephrotic Syndrome"[Mesh]) AND "Vaccines"[Mesh] AND "Child"[Mesh], limited to 15 years with exclusion of articles written in other languages than English.

`

| Study | Patients | Clinical Settings | Median Age (years) | Efficacy | Safety |
| --- | --- | --- | --- | --- | --- |
| 2002 Alpay et al | 20 patients  22 healthy controls | VZV vaccination in INS children  Prospective interventional study with a 2-year follow-up | 4.7+/- 1.8 | Not significantly different seroconversion rate between patients and controls. | 3 patients with mild chickenpox during the following month. 1 patient with a varicelliform rash.  1 child with relapse 3 weeks after vaccination. |
| 2003 Abeyagunawardena et al | 106 Patients with INS | Meningococcal C conjugate vaccine in INS children.  Retrospective cohort study | NA | Not studied | Significant increase of relapse rate during the year post-vaccination compared to the year pre-vaccination: relative incidence of 1.52 (95% CI 1.10–2.11; p=0.009). |
| 2004 Hakan M. Poyrazog ̆lu  et al | 19 children with INS  10 healthy controls | Antibody response to influenza A vaccination in INS children  Prospective interventional study with a 6-month follow up | Patients: 6.6 ± 2.3 years  Controls: 9.2 ± 0.9 years | Not significantly different antibody titers between the 2 groups 1 month after immunization.  No decrease in Ab titers and proportion of children with protective antibody titers 6 months after immunization. | No adverse reaction  Frequency of relapse before and after vaccination was not different. |
| 2007 Taylor et al | 53 patients | Meningococcal C conjugate vaccine (MCCV) in INS children.  Retrospective cohort study | 4.9 years | Not studied | No increased risk of relapse of INS after vaccination with MCCV |
| 2008 Ulinski et al | Group 1: 30 patients with INS at disease onset  Group 2: 13 patients with INS in remission  Group 3: 25 non-vaccinated patients with INS | Serological response to 23-valent pneumococcal vaccine (PV) in INS patients at disease onset on high-dose prednisone | Group 1: 5.3 (2.0–16.0) years  Group 2: 4.5 (2.0–9.0) years  Group 3 : not specified | Not significantly different initial serological response and development of serum titers over time between Group 1 and 2..  No pneumococcal infection | Immediate vaccination at disease onset did not increase disease activity |
| 2011 Liakou et al | 33 patients with INS  Group A: low dose of prednisone (<1mg/kg/j)  Group B: immunosuppressive drugs  16 healthy controls | Pneumococcal-conjugate vaccine (PVC7) in INS patients  Follow up  Prospective interventional study with a 12-14-month follow-up | Group A: 8.1 ± 2.2  Group B: 11.4 ± 3.6  Control group: 13.7 ± 3.7 | Sustained protective Ab titer for ≥5 pneumococcal serotypes in 86%, 94% and 100% in Group A, B and controls)  Inferior magnitude and persistence of response to some serotypes in patients compared to healthy subjects 🡪 Need for revaccination  No invasive pneumococcal infection | No increased risk of relapse (RR=0.80)  Similar local and systemic reactions |
| 2013 Yildiz et al | 41 patients with INS  30 healthy controls | Hepatitis B virus vaccination in INS patients | 7.5 ± 1.8 | Significantly lower seroconversion rate at 15 months in the full-dose steroid users (n=14) compared to the controls (p=0.009) | Increased relapse rate (0.12% pre-vaccination versus 0.4% post vaccination p=0.002) |
| 2013 Liakou et al | 29 patients with INS  Group A: low dose of prednisone (<1mg/kg/j)  Group B:  immunosuppressive drugs | Pneumococcal-conjugate vaccine booster (PVC7) in INS patients  Prospective interventional study with a 6-month follow-up | Group A: 5.0 ± 1,5  Group B: 3.4 ± 1.4 | No invasive pneumococcal infection | No increased risk of relapse |
| 2013 Mantan et al^41^ | 75 patients with INS.  42 with steroid-resistant nephrotic syndrome (SRNS)  33 with steroid-sensitive nephrotic syndrome (SSNS) | Cross-sectional study | 6.9 (1-18) | 48 % patients had anti-HBs protective titers  63.6 % children with SSNS had protective titers  35.7 % in SRNS subjects  (p=0,016) | Not Studied |
| 2016 Pittet et al | 42 patients with INS | 13-valent pneumococcal conjugate vaccine in INS patients.  Prospective cohort study with a 12-month follow-up | 7.7 (5.1–11.4) | No pneumococcal sepsis, peritonitis or biologically proven pneumonia  High immunogenicity, regardless of the treatment even after 1 year. | Similar local and systemic reactions  No serious adverse event  19% of NS relapse during the following year compared to the 40% in the year preceding inclusion. |
| 2018 Kamei et al | 60 patients with INS in remission and immunosuppressive treatment | Live attenuated vaccines: measles, varicella, mumps, rubella.  Prospective monocentric study with at least 12 months follow-up | 8 (1-24) | Seroconversion rates for measles and rubella similar to the general population (90%-95%)., Seroconversion rates for varicella (61.9%) and mumps (40.0%) lower than the the general population (80%-85%) | No serious adverse effect |
